# Algebraic Multi-Layer Network: Key Concepts

**DOI:** 10.3390/jimaging9070146

**Published:** 2023-07-18

**Authors:** Igor Khanykov, Vadim Nenashev, Mikhail Kharinov

**Affiliations:** 1 Laboratory of Big Data Technologies for Sociocyberphysical Systems, St. Petersburg Federal Research Center of the Russian Academy of Sciences, 14 Line V. O. 39, 199178 Saint Petersburg, Russia; igk@iias.spb.su; 2 Laboratory of Intelligent Technologies and Modelling of Complex Systems, Institute of Computing Systems and Programming, Saint Petersburg State University of Aerospace Instrumentation, 67 B. Morskaia St., 190000 Saint Petersburg, Russia; nenashev@guap.ru

**Keywords:** image processing, cluster analysis of big data, Ward’s pixel clustering, Sleator–Tarjan dynamic trees

## Abstract

The paper refers to interdisciplinary research in the areas of hierarchical cluster analysis of big data and ordering of primary data to detect objects in a color or in a grayscale image. To perform this on a limited domain of multidimensional data, an NP-hard problem of calculation of close to *optimal* piecewise constant data approximations with the smallest possible standard deviations or total squared errors (*approximation errors*) is solved. The solution is achieved by revisiting, modernizing, and combining classical Ward’s clustering, split/merge, and K-means methods. The concepts of objects, images, and their elements (*superpixels*) are formalized as structures that are distinguishable from each other. The results of structuring and ordering the image data are presented to the user in two ways, as tabulated approximations of the image showing the available object hierarchies. For not only theoretical reasoning, but also for practical implementation, reversible calculations with pixel sets are performed easily, as with individual pixels in terms of Sleator–Tarjan Dynamic trees and cyclic graphs forming an Algebraic Multi-Layer Network (AMN). The detailing of the latter significantly distinguishes this paper from our prior works. The establishment of the invariance of detected objects with respect to changing the context of the image and its transformation into grayscale is also new.

## 1. Introduction

Modern computer vision is a set of heuristic models and specific solutions for predetermined types of images (remote, medical, technical, etc.) with apriori known objects-of-interest (underlying surfaces, cell nuclei, elements of chips, etc.) that are detected to solve current production problems in real time. At the same time, the theory of adequate primary image processing [1,2], which substantiates object detection methods and formalizes the very concept of an image, objects in an image, and superpixels, is still in its infancy. This is why papers on computer vision are replete with pictures which could be dispensed with in a proper description of the computational formalism available to a computer.

Meanwhile, it is the detection of objects at the primary stage of processing that is the stumbling block in computer vision and image recognition. Typically, automated image recognition begins with *segmentation*, i.e., the division of the image into connected segments, and the connectivity of the segments is not only achieved as a result, but is also maintained in the process of calculations. Compared to conventional image segmentation, more general pixel clustering [3,4,5,6] allows better calculation of the resulting segmentation. However, for equal programming effort, pixel clustering takes much longer. This is true for *N*-pixel image segmentation without real optimization of the quality criterion, namely, the standard deviation σ or, equivalently, the approximation error $E=3N\sigma^{2}$, since the problem of minimizing the approximation error *E* in image segmentation is easiest to solve by starting with optimal pixel clustering [7].

As for the theoretical requirements for segmentation, they are formulated in [8] on the basis of a generalization of the segmentation model [9,10] and similar others, but have not yet led to a generally accepted interpretation. Regarding the basic principles of segmentation, Georges Koepfler in [8] states the following:

1. We admit the possibility of a universal boundary detection device, definable and analyzable independently from the kind of channels to be used in the texture discrimination problem (see [11,12]). This allows us to begin to obtain a mathematical understanding of the segmentation problem by considering gray-level segmentation;
2. An algorithm for boundary detection must be scale- and space-invariant. This means that multiscale segmentation algorithms, invariant by rotation and translation, should be considered;
3. The last point is what we shall call the comparison principle. Given two different segmentations of a datum, we shall be able to decide which of them is better than the other. This implies the existence of some ordering on the segmentations which is reflected by some real functional *E* such that, if $E\left(K_{1}\right)<E\left(K_{2}\right)$, then the segmentation $K_{1}$ is “better” than the segmentation $K_{2}$.

For comparison, according to our version of the interpretation and development of the above principles, they are expressed as follows:

1. We consider grayscale images as a special case of color ones and admit the possibility of a unified multi-valued detection of objects defined only by the image, regardless of processing methods. Adequate segmentation expresses an inherent property of an image that distinguishes an image from arbitrary data if the segmentation is achieved as a result of pixel clustering. We do not allow objects to be defined on specific examples and provide adaptive top-down processing, in which the texture discrimination problem loses its specificity;
2. Our experience confirms that object detection and image segmentation via pixel clustering must be multiscale and commute with image scaling. Moreover, to better account for image ambiguity, we represent the image as a set of approximation hierarchies. The condition of spatial invariance is also satisfied, since, in our approach, the clustering of pixels is not affected by their geometric distribution;
3. Of the two image approximations in the same number of colors, the approximation with the less approximation error *E* is considered to be better, which is quite consistent with visual perception. In addition, a characteristic pattern for images has been established, which is that, with an increase in the number of colors and a decrease in the approximation error of optimal approximations, the negative derivative $\frac{\partial E}{\partial g}$ of the approximation error *E* with respect to the number of colors *g* increases.

In the original, the idea of implementing the G. Koepfler’s principles was to build a minimization function consisting of at least two additive terms so that, depending on the number of clusters or another “scaling” parameter, the minimum was achieved for the desired segmentation. The first term is the approximation error *E*, and the second is the total length of the segment boundaries [9,10]. However, in [13] (and our experience), compared to the approximation error *E*, the additive second term in the functional often has an insufficient effect on the calculation results. Therefore, we have omitted the additional term and identified the functional *E* with the “pure” approximation error.

It is important to note that, in the latter case, the optimization problem retains a nontrivial meaning if the approximation errors *E* are counted not from zero, but from optimal values depending on the number of clusters, i.e., on the number of colors in a given approximation of the image.

Thus, guided by the general principles of [8], we associate their implementation with the well-known problem of calculating optimal image approximations using classical methods of cluster analysis. It is in the solution of the classical problem by only classical methods that the novelty of our approach lies, since the real-life *E* minimization still remains insufficiently studied. Contributing in part to the lack of attention to image approximation quality optimization is [14]’s assertion that standard deviation σ and approximation error $E=3N\sigma^{2}$ are inconsistent with natural visual perception, which has become a popular stereotype in image processing. In fact, this is not the case if cluster analysis is applied in strict accordance with the classical guidelines [15,16].

Exact minimization of the approximation error *E* for arbitrary data is NP-hard (see [17] and subsequent works, most fully listed in [18]). Although the NP-hardness of the problem does not exclude an approximate solution within a formally limited subject area [19], there are not enough enthusiasts who are ready to solve this for Full HD images. As far as we know, the entire range of optimal approximations has been established for only one standard image [20]. In cluster analysis, the effective minimization of the approximation error for millions of data elements is hindered by the lack of a formal description of a specific data domain, as well as limitations caused by insufficiently efficient work with hierarchies using dendrograms [21,22,23,24]. To perform reversible calculations [25,26] with optimal and nearly optimal image approximations, as well as to generate, store, and transform their hierarchical sequences adaptively to the image, it is necessary to use Sleator–Tarjan Dynamic trees [27,28] instead of *ordinary trees* (dendrograms). Sleator–Tarjan Dynamic trees, supplemented with cyclic graphs in the Algebraic Multi-Layer Network data structure, make it possible to obtain truly optimized image approximations using limited computer memory and in a reasonable time.

The main purpose of this paper is to describe the key points of the AMN software implementation, as well as highlight the developed principles for object detection.

The main contributions of the paper to image processing and cluster analysis lie in the proposed elements of the theory of hierarchical clustering of big data:

- Structural definitions of the concepts of an image, its elements, (superpixels) and objects in the image;
- Proposition about the convexity of the sequence of the minimum possible approximation errors in a different number of colors;
- Mathematical substantiation of the system of methods for minimizing *E*, including the correction of the K-means method and the rules for their joint application;
- Apparatus for reversible calculations with pixel sets in terms of the Algebraic Multi-Layer Network based on Sleator–Tarjan Dynamic trees and cyclic graphs.

The rest of this study is arranged as follows.

Section 2 describes the research methodology, formalizes the basic concepts and formulation of the combined approximation–optimization problem, proves the existence of solution by Ward’s pixel clustering, outlines a target software implementation of the primary structuring and ordering of image data, and derives the main tuning parameter.

Section 3 describes the main shortcomings of the three original cluster analysis methods when applied to big data, and suggests how to modernize these methods to effectively apply them together.

Section 4 introduces the concept of an Algebraic Multi-Layer Network.

Section 5 is the main one in the paper. It describes the key points of programming the generation and conversion of AMN. This section is intended primarily for programmers.

Section 6 provides some general remarks that motivate the development of object detection based on advanced optimization of image approximations. It describes the current state of development and implementation of the discussed pixel clustering methods. The conclusion is made about the relevance of modernizing methods for minimizing the approximation error in widely used software tools.

Section 7 demonstrates some of the image experiments available without time-consuming pre-programming. In this section, for the first time, so-called Dynamic Superpixel Table is introduced to represent the output of image structuring. In addition, a solution to the problem of detecting objects using pixel clustering invariant to changes in the image content and converting into a grayscale representation has been started. A generalization of the Euclidean color space to the case of four dimensions is mentioned.

The final section, Section 8, highlights future research directions. In this Section, an additional mechanism for accelerating calculations due to pixel decimation is outlined as the next step in the study.

## 2. An Approach to Detecting Object Hierarchies Using Pixel Clustering

Unlike conventional papers on the detection of specific objects, representing the image as a matrix of arbitrary pixels, we describe the image by a sequence of optimal approximations, which are ordered by the number of colors *g*, the approximation error *E*, and the derivative $\frac{\partial E}{\partial g}$ of the approximation error *E* with regard to the number of colors *g*. We represent objects as being nested within one another and described with a binary hierarchical approximation sequence that is also ordered by *g*, *E*, and $\frac{\partial E}{\partial g}$. Since the image contains different objects, it appears as an ordered *polyhierarchical* structure, which is not itself hierarchical, but can be approximated by one or another hierarchical sequence corresponding to objects.

The research methodology for the proposed primary structuring and ordering of the data contained in the image is being developed within the framework of Descriptive Approach to Image Processing [29], which states “that the image representation is transformed from the original form into a form that is convenient for recognition (i.e., into a model)”. At the same time, our goal is to obtain a simplified solution that is implementable as a certain computer program or software package. Unlike purely engineering solutions, we prefer to automate adequate image processing first, and only then speed up calculations, without losing the quality of the results, especially since automatic processing can be significantly accelerated by running several copies of the program on several computers.

The developed theoretical principles for detecting objects in an image by classical methods of minimizing the approximation error *E* are expressed by the following provisions:

- The approximation error *E* is taken as a quality criterion for image approximation. To actually minimize the error *E*, image segmentation is performed through pixel clustering;
- The convexity of the minimal approximation errors sequence of the *optimal* image approximations in 1,2,… colors serves as a criterion for the correspondence of the input data to the computerized model;
- The concepts of objects, images, and their elements, called superpixels, are treated and implemented as functions of image only;
- The invariance of calculations with respect to the linear transformation of pixel numbers or their intensities, including transformation from positive image to negative, is supported. The results of clustering are not affected by changing the geometric placement of pixels. These properties make commutative pixel clustering and image scaling by pixel duplication;
- Calculations are performed according to the correctly formulated statement of the approximation–optimization problem in two-dimensional ordering of image approximations to choose desired object hierarchy.

**Figure 1:**
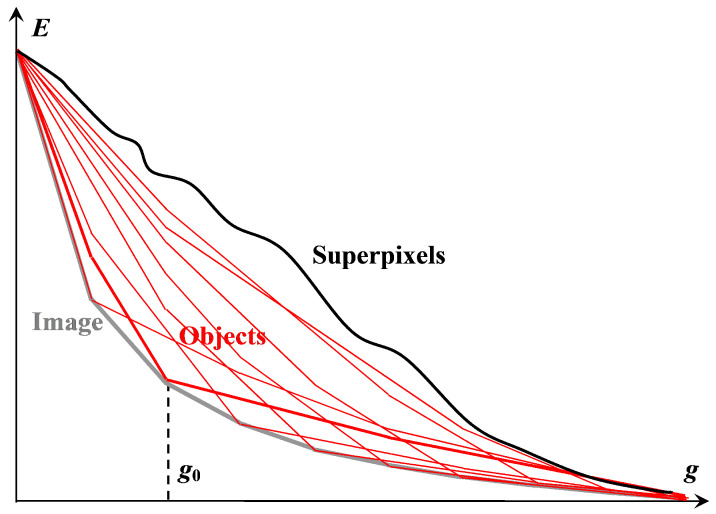
The existing multi-valued solution of the problem of image hierarchical approximation, achievable by Ward’s pixel clustering. The lower gray convex curve describes $E_{g}$ sequence of optimal image approximations. The upper non-convex black curve describes errors $E_{g}$ of image approximations by superpixels constituting some irregular hierarchical sequence. The remaining red convex curves describe $E_{g}$ sequences of hierarchical image approximations, each containing at least one optimal approximation in a corresponding incrementing number of colors.

The listed principles are illustrated in Figure 1, which explains the following:

- The formulation of the combined approximation–optimization problem;
- Structural definitions of an image, objects in an image, and superpixels;
- Proof of the existence of a solution by Ward’s pixel clustering [15,16,18,21];
- Derivation of the main tuning parameter $g_{0}$;
- The way of ordering $N^{2}$ output image approximations, which are presented to the user as a result of the primary image processing, where *N* is the number of pixels in the image.

We start the discussion of Figure 1 with some definitions.

A set of pixels is considered *structured* if it is represented by a sequence of approximations in g=1,2… colors, described by a convex sequence of approximation errors.

*Object* consists of nested ones and is ambiguously represented by a binary hierarchy of piecewise constant image approximations that contain at least one optimal image approximation in $g_{0}$ colors. The hierarchy of objects, depending on the number *g* of colors, is described by a convex sequence of total squared errors $E_{g}=3N\sigma_{g}^{2}$, where *N* is the number of pixels and σ is the standard deviation.

An *image* is a polyhierarchical structure, which, depending on the number *g* of colors, is represented by a sequence of overlapping optimal image approximations and is described by a convex sequence of total squared errors $E_{g}=3N\sigma_{g}^{2}$.

*Superpixels* are generated by the intersection of successive optimal image approximations from the restricted approximation series in such a way that, when merged, they provide error-free reproduction of this series of optimal approximations and, with a variable number of accounted optimal approximations, form an irregular hierarchy described by a non-convex (sinuous) dependence $E_{g}$ of *E* on *g*.

Thus, the image and objects are structured, while the superpixels are unstructured, in accordance with our convention for the term “structure”.

Figure 1 shows *approximation errors*, i.e., total squared errors $E_{g}$, for image approximations in g=1,2,…N colors. The lower gray convex curve describing the minimum possible approximation errors of the *optimal* color image approximations is simulated by the upper red convex curves describing the approximation errors of the image approximation hierarchies.

The convexity of the lower gray curve for optimal approximation errors is a model assumption about an inherent property of the image, which is verified experimentally. The convexity of the upper red curves of approximation errors of hierarchical approximation sequences is achieved by construction. The upper red curves in Figure 1 do not merge with the lower gray curve because the pixel clusters of optimal approximations overlap with each other and the optimal approximation sequence is not hierarchical.

It is easy to convey that any hierarchy of image approximations can be obtained by Ward’s method if the optimal image approximations are known. To obtain an approximation hierarchy containing the optimal approximation in $g_{0}$ colors, it suffices to perform the following:

- To calculate the nested hierarchy of pixel clusters within each of the $g_{0}$ clusters of a given optimal approximation by means of Ward’s method;
- To re-sort the order of cluster merging in ascending approximation error increment without changing the clusters themselves;
- To complete the resulting hierarchy using Ward’s pixel clustering within the whole image.

Figure 1 describes $N^{2}$ image approximations, which are provided to select from them a desired hierarchy of *N* approximations and set the tuning parameter $g_{0}$.

Obviously, neither matrix representation, nor even coding in terms of ordinary trees or dendrograms generating a set of extra nodes, is suitable for encoding $N^{2}$ image approximations in RAM in order to present any desired approximations to the user in the form of fragments of the so-called Dynamic Table of size N×N [7]. In Dynamic Table, hierarchical sequences of image approximations, described by red convex curves in Figure 1, are placed in columns from top to bottom in ascending order of number of colors *g*, and the columns are ordered from left to right in ascending order of parameter $g_{0}$. In this case, the sequence of diagonal approximations is described by the limit lower gray convex curve and constituted by approximations with the approximation errors that are minimal within the rows of Dynamic Table. In image processing, a two-dimensionally ordered Dynamic Table of hierarchical image approximations provides a visual setting for pixel clustering.

For efficient computations, Sleator–Tarjan Dynamic trees are indispensable, since they are generated on a given node set located in *N* pixel coordinates. In general cluster analysis, hierarchical clustering is usually described using ordinary trees generated as dendrograms [21,22,23]. This makes it difficult to study Ward’s method for big data. Because the adaptive image approximation hierarchy is initiated in a bottom-up strategy, all $N^{2}$ approximations must be encoded in RAM, which seems difficult to achieve with ordinary trees due to the generation of additional nodes. Therefore, to generate, store, and transform $N^{2}$ image approximations in RAM, it is better to master Sleator–Tarjan Dynamic trees.

The target computer program for structuring and ordering data for detecting objects in an image is characterized by the following:

- The content of the input image is not limited in any way;
- A hard-to-formalize procedure of program learning is not provided. The problem of using a priori information is bypassed. To take into account the previous processing experience, an input enlarged image with attached additional images of objects-of-interest is used, which makes it possible to dispense with the analysis of features and identification when objects are detected by the advanced pixel clustering methods [30];
- Instead of program learning, for the convenient processing control, tuning parameters are introduced, which are determined by the problem statement. The main tuning parameter is equal to the number $g_{0}$ of colors in that optimal image approximation, which is most suitable for hierarchical approximation of objects-of-interest with either unions or parts of $g_{0}$ pixel clusters.
- The program outputs $N^{2}$ image approximations ordered in a two-dimensional Dynamic Table [7]; Hierarchies of the image approximations occupy the Dynamic Table columns, ordered so that the optimal image approximations are on the diagonal.

## 3. Three Classical Clustering Methods and Their Modernization

This section provides a brief summary on upgrading three methods for minimizing the color image approximation error: Ward’s clustering, split/merge, and K-means methods, since they are detailed in our preceding paper [7].

Ward’s method in cluster analysis is usually treated as the simplest algorithm for generating a *structured* hierarchical approximation sequence described by a convex sequence of approximation errors [15,16,17,18,21,22,23,24]. The characteristic ambiguity of an image representation by approximation hierarchies is not given due attention. In the case of big data, multi-iteration calculations lead to different approximations of the image in a limited number of colors due to the variability in the input data, as well as due to changes in the scanning order of data items and ways to speed up calculations to overcome excessive computational complexity.

In image processing, Ward’s pixel clustering is rare, where, on the morrow of the papers on cluster analysis [17,18], it is predominantly applied to calculate the initial clustering for the K-means method [31,32]. In some special cases of processing laboratorial images, Ward’s pixel clustering is rated as superior to other methods, usually successors to K-means [33,34]. According to our data, the dependence of Ward’s clustering on the algorithm for its implementation has so far remained unnoticed either in cluster analysis or in its applications for detecting objects in an image.

Meanwhile, the “instability” drawback, i.e., a property inherent to the image (Figure 1), easily turns into an advantage of the method if Ward’s clustering is supplemented by minimizing the approximation error *E* for a given color range, say, from 1 to 20. The easiest way to carry this out is to run Ward’s method, say, 100 times and, from the resulting hundred approximation hierarchies, select those 20 that contain approximations with the minimum error for each considered number of colors. As a result, using only Ward’s original method, we obtain 20 optimal (or close to optimal) approximations that better represent the image, since the optimal approximations depend only on the image and do not depend on the generation algorithm.

Modernized versions of split/merge and K-means methods are needed to speed up calculations and avoid repeating the execution of Ward’s method many times.

For a known optimal image approximation, the hierarchy containing this approximation is generated by Ward’s method applied to image parts, as described in the previous section. Image processing in parts by Ward’s method provides faster computations because, due to the reduction in the number of pixels, the computational complexity drops quadratically, while linearly increasing along with the increasing number of image parts. If the optimal approximation of the image is not known in advance, then *N* pixels can be divided into subsets in any way. However, to then obtain an approximation hierarchy without violating the convexity of the corresponding approximation error sequence, it is necessary to process the structured image parts by the so-called CI (Clustering Improvement) method before iteratively merging the structured image parts to complete the hierarchy [7].

The CI method is a trivial *greedy* method for iteratively reducing the approximation error *E* as much as possible by dividing one pixel cluster into two, followed by merging a pair of clusters so that the number of colors in the refined approximation remains unchanged. This is a split/merge method that does not change structured hierarchical image approximations such as those obtained by Ward’s original method. Similar to other analogous split/merge methods, the CI method uses a pre-generated binary hierarchy of image approximations. Conventional split/merge methods typically use a non-adaptive pyramidal hierarchy [35,36]. In contrast to these, the CI method utilizes an adaptive hierarchy, which, due to reversible calculations, is built in both bottom-up and top-down strategies. It is important that the specified binary hierarchy is supported in terms of Sleator–Tarjan Dynamic trees, which, compared to ordinary trees, simplify and speed up calculations, and also reduce the consumed computer memory. Initially, the CI method was invented and studied as an independent heuristic method for conventional image segmentation [37]. The results of segmentation improvement turned out to be very promising and all the more spectacular; the worse approximation was chosen for improvement. As applied to pixel clustering, the CI method fits better with the theoretical *E* minimization scheme. In this case, the CI method is even simplified, since, when splitting a cluster into two, it is not required to maintain the connectivity of the segments. As such, there is no doubt that, for image segmentation through pixel clustering, the CI method will be quite useful for solving applied problems.

According to our logic of combining the classical methods of minimizing the approximation error *E* into a system, the CI method and Ward’s clustering together provide efficient acquisition of image approximation hierarchies described by a convex sequence of errors *E*. To obtain an ordered sequence of hierarchies, as in Figure 1, it is necessary to convert such hierarchies from one to another. The desired method is obtained by modifying the well-known K-means method [17,18,31,32,33,34,38,39,40,41,42,43].

From the point of view of efficient *E* minimization, the K-means method has the following two main disadvantages:

- Strictly speaking, the K-means method, as is usually stated, only “tends” to minimization, but in general does not minimize the approximation error *E*, since it uses an unreasonably rough formula for error increment ΔE;
- The calculation of average pixel values in the K-means method hinders the minimization of the *E* error, since it entails staleness errors due to data deterioration.

To verify the validity of the first statement, it is enough to analytically derive the K-means method, which, in fact, reduces to reclassifying pixels from one cluster *i* to another cluster *j*, using the formulae for the increment ΔE of the approximation error *E* caused by the merging or inverse splitting of clusters from $n_{i}$ and $n_{j}$ pixels:

(1) $$\Delta E_{merge}\left(i,j\right)=\frac{n_{i}n_{j}}{n_{i}+n_{j}}{∥I_{i}-I_{j}∥}^{2}\;\Rightarrow \;E_{split}\left(i\cup j\right)=-E_{merge}\left(i,j\right),$$

where $I_{i},I_{j}$ denote the three-dimensional average pixel values within the clusters i,j, and $∥I_{i}-I_{j}∥$ denotes the Euclidean distance.

From the expressions in (1), it is easy to derive the formulae for the approximation error increment $\Delta E_{correct}$, accompanied with the reclassification of *k* pixels from the cluster *i* to the cluster *j*:

(2) $$\Delta E_{correct}=k\left(\frac{n_{j}}{n_{j}+k}{∥I_{j}-I_{k}∥}^{2}-\frac{n_{i}}{n_{i}-k}{∥I_{i}-I_{k}∥}^{2}\right)$$

or, in simplified form:

(3) $$\Delta E_{correct}^{rough}=k\left({∥I_{j}-I_{k}∥}^{2}-{∥I_{i}-I_{k}∥}^{2}\right),$$

where $I_{k}$ is the average value of *k* pixels, which are excluded from the *i*-th cluster and assigned to the *j*-th cluster.

The exact criterion for reclassifying *k* pixels is the condition $\Delta E_{correct}<0$. However, K-means instead uses the rough condition $\Delta E_{correct}^{rough}<0$, which is obtained by eliminating (from Expression (1)) the multiplier that depends on the pixel numbers $n_{i}$ and $n_{j}$. Obviously, if this multiplier is ignored, this violates the correctness of applying the reclassification of pixel sets along with the reclassification of individual pixels.

The discrepancy between the Formula (1) used by Ward’s method and the Formula (3) used by the K-means method is the major hurdle to use them together to effectively minimize the *E* error, according to [17,18] and a number of other works. As for the calculation of cluster centers in the K-means method, it is useful to exclude it, as well as calculations of other unnecessary intermediate data, if only in order to not encounter empty clusters, as in [43]. The latter is carried out in the greedy K-meanless method [44], providing reclassification of pixel subsets from cluster to cluster in accordance with the condition of maximum reduction of the approximation error, which is estimated either by calculating the functional *E* itself or by calculating its increment ΔE using (2).

The use of the exact Formula (2) for ΔE and the elimination of intermediate calculation of the pixel cluster centers increases the efficiency of minimizing the error *E* without affecting the speed of computer calculations. Computing in terms of Sleator–Tarjan Dynamic trees unsurpassedly increases the speed and reduces the computer memory consumption, especially when using the proper data structure, which is discussed in the next two sections.

## 4. Scheme of Algebraic Multi-Layer Network (AMN)

The image model described in [7] and outlined here is transparent and is based on the classical cluster analysis methods. Nevertheless, its software implementation is non-trivial, as it demands mastering an equivalent calculation model which would allow one to work with superpixels and other pixel sets as easily as with the source pixels of the image.

The pixel clusters in question constitute a so-called *adaptive* hierarchy, i.e., they are calculated from the image in merge mode and are not pre-restricted in any way. Each hierarchy contains *N* image approximations and 2N−1 pixel clusters which are stored and processed in-memory. For the millions of pixels in the image, it is obviously impossible to ensure if approximations are coded in the conventional matrix format. However, the effect of multiple representation storage is achieved by calculations via a certain network. A specific requirement to such a network is to support reversible calculations [25,26], which can be implemented in two ways:

- As unlimited rollback of calculations for any number of steps;
- In terms of reversibility of cluster merge operation, which allows one to modify and optimize calculations performed in reverse order.

**Figure 2:**
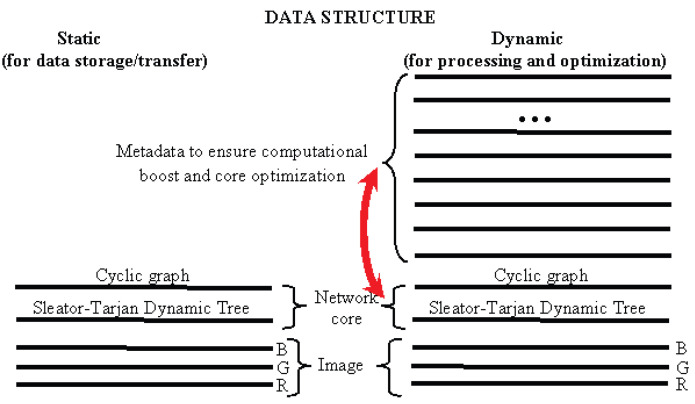
Scheme of the Algebraic Multi-Layer Network.

As a specific technical solution, this paper introduces an Algebraic Multi-Layer Network (AMN) which provides memory and time optimization of reversible calculations but does not require additional learning and is not intended to imitate intelligence, in contrast to the currently common Artificial Neural Networks. The network under consideration is called *algebraic*, as it is obtained by the merge of cyclic and acyclic graphs (trees). This network is a *multi-layer* one, as it is defined in pixel coordinates by arrays of the same length of *N* items (Figure 2).

Figure 2 illustrates the Algebraic Multi-Layer Network, which is “thrown over” the pixels of the image. It shows a stack of five images each containing *N* pixels on the left and an extended stack of, say, 50 images on the right. In a computer, pixels are ordered linearly. Therefore, instead of two-dimensional images, linear arrays are shown. The trick is that, in this amount of memory for an image of a million pixels ($N=10^{6}$), a billion approximations (stack of $N^{2}=10^{12}$ images) are encoded and available on-line. This seems amazing, but only for those who confuse Sleator–Tarjan Dynamic trees with ordinary ones.

During storage and transfer steps, the network contains only two graphs: a tree and a cyclic graph, which, together with the image pixels, encode the hierarchy of *N* image approximations.

As part of general data structure, the above-mentioned network of two graphs is called the network core.

In the process of detecting objects in an image of *N* pixels by the core network, metadata are generated, i.e., several more dozens of graphs, pointer systems, numerical arrays, and other extra components of *N* items. Metadata are calculated in one pixel scanning step and support the whole complex of reversible calculations, including the network composition and optimization (double-arrow in Figure 2), as well as high-speed operations with pixel clusters.

For some clarification, it seems appropriate to associate graph-based “neurons” of AMN with some subset of neurons in Natural Neural Networks.

In AMN, the “neurons” at each layer perform the same actions: store a number, point out to the “neurons” of their own or another layer, or they are pairwise added together according to some pointers, for example, according to the edges of a Sleator–Tarjan Dynamic tree.

In Figure 2, the color image components are included in the three lowest layers of “neurons”. The next two layers constitute a Sleator–Tarjan Dynamic tree [27,28] and a cyclic edge interleaving graph, which are built on a set of pixel coordinates. Metadata include a layer with the weights $H\equiv \left|\frac{\partial E}{\partial g}\right|=\left|\Delta E_{split}\right|$ of the edges in a Sleator–Tarjan Dynamic tree. A feature of AMN is that the edge weights are calculated automatically. Another feature of AMN is that the “neurons” in the layer, although they perform the same operation, are able to act collectively. In this case, co-operative routine operations on fixed length data arrays provide an efficient programmatic and algorithmic solution to the optimization problem, but the main advantage of AMN is that, for a specific optimization problem of structuring images and objects, a solution is provided that uses classical methods of cluster analysis.

High-speed calculations are expressed in terms of AMN by simple programs which perform routine operations on specialized “neurons” layers to describe reversible merge and split of pixel clusters while optimizing the hierarchy of image approximations.

At a low level of programming, AMN (Figure 2) is formed and transformed by two mutually reversal program modules:

- Program for pairwise merging of pixel clusters;
- Program to split a pixel cluster into two.

To execute the first program, one must specify a pair of clusters to be merged. Another program splits given cluster into two clusters, by merging what was obtained. If splitting the clusters in two is performed in a different order than the reverse order, then AMN state is modified, which is used to reduce the approximation error in the image approximation hierarchy optimization algorithm.

## 5. The Hornbooks of Calculations in Terms of Cyclic and Acyclic Graphs

In the clustering model for image segmentation and object detection, discussed here, the hierarchy of approximations is encoded with acyclic graphs, or trees. Nevertheless, the interpretation of trees in the model differs from the stereotypical concept of conventional trees [17,45], which are often called dendrograms in the image processing domain. Instead of conventional trees, our model uses so-called Sleator–Tarjan Dynamic trees [27,28], which are much more memory-efficient as compared to conventional trees. In addition, Sleator–Tarjan Dynamic trees support reversible operations with pixel clusters and provide processing of any binary hierarchy of pixel clusters, comparable in speed to the processing of individual pixels.

Contrary to conventional trees, Sleator–Tarjan Dynamic trees are as follows:

- They are constructed on the sets of pre-defined nodes without generating extra nodes;
- They are placed *over* pixels (in their coordinates), so they are clustered together with pixels;
- They set a binary hierarchy of nodes by means of an irregular tree.

Sleator–Tarjan Dynamic trees are mapped to image pixel clusters. In this case, the image pixels are considered to be linearly ordered in some scan order. First, each pixel is assigned to a specific cluster and mapped to a tree consisting of a single root-node that is encoded by a node pointer pointing to itself. An iterative merging of the pixel clusters is then performed, which is encoded by the corresponding merging of the trees. In a tree merge operation, an edge pointer is set from a pixel with a higher number to a pixel with a lower number.

**Figure 3:**
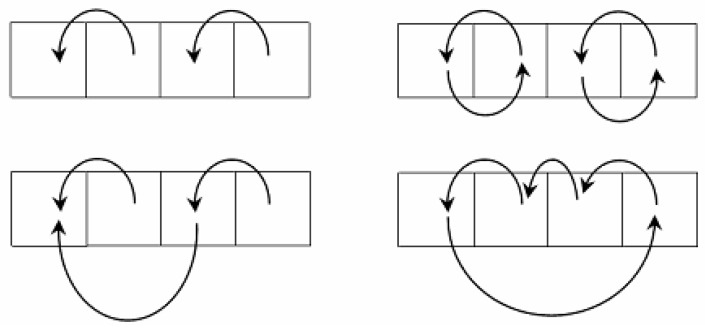
Tree merge (**left**) and cyclic graph merge (**right**).

In the mathematical structure of Algebraic Multi-Layer Network (AMN), the Sleator–Tarjan trees are augmented by cyclic graphs that are initiated by pointers pointing to themselves, similar to Sleator–Tarjan Dynamic trees. Conversely, unlike the merging of trees, when a pair of cyclic graphs is merged, the corresponding pair of definite nodes exchange pointers with each other (Figure 3).

Figure 3 illustrates a tree merge and a cyclic graph merge. The top row shows trees (on the left) and cyclic graphs (on the right) before the merge, and the bottom row shows them after the merge. In a tree merge, the major root-node is assigned with a pointer to a minor root-node. In a cyclic graph merge, cyclic graph nodes swap pointers, which account for the root-nodes of the trees.

Figure 3 shows the principle of Sleator–Tarjan tree and cyclic graph merges. Owing to cyclic graphs chosen for an example, pixel clusters coordinates lists are available at each step of processing. Additionally, calculation of any cluster characteristics is provided as needed via scanning of pixels. Using cyclic graphs which bind the pixels in clusters, as in Figure 3, in addition to Sleator–Tarjan trees computation, it is convenient in the process of computation to obtain *straightened* trees in which all the nodes point to the root-node.

Current data, accessible without pixel scanning, such as the number of pixels and other additive characteristics, are stored in the root-node of an enlarged tree and are not modified until the pixel cluster represented by this root-node is further extended. For the pixel cluster under consideration, static data, which are retained even after the extension step, are stored at the edge address, i.e., at the address of the node from which the edge originated. A typical example of static data is the set of values of heterogeneity parameter $H\equiv \left|\frac{\partial E}{\partial g}\right|=\left|\Delta E_{split}\right|$, where such values are used in networking computations as weights attributed to edges.

**Figure 4:**
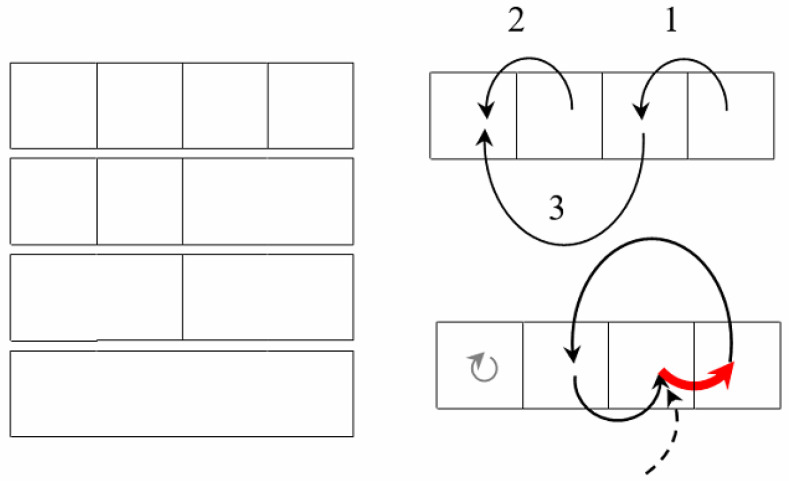
Reversible coding of the partition hierarchy by means of the network core. On the left the segmentation sequence is shown for an image of four pixels encoded by a pair of graphs presented on the right. In the upper right corner the appropriate Sleator–Tarjan Dynamic tree is shown. In the lower right corner, the bold red arrow indicates the pointer to an edge of a tree which was the first to be set. The dashed arrow is an external pointer to this arrow. The next black arrow shows a pointer to the edge, which was set second, etc.

Reversibility of pixel cluster merge is provided by the network core Figure 4, which is established as follows:

- Sleator–Tarjan tree (upper right-hand corner);
- Cyclic graph of edge interleaving in Sleator–Tarjan tree (lower right-hand corner);
- Pointer to the last edge of a cyclic graph indicating its origin point (dotted arrow).

For the sake of clarity, the edges in the upper right-hand picture are numbered in the order of setting.

On the left part of Figure 4, for an image of N=4 pixels, a hierarchy of partitions into clusters of 4, 3, 2, and 1 pixels is shown. The right part of Figure 4 illustrates the encoding of the specified hierarchy in two ways. Figure 4 at the top right shows the memorization of a hierarchy using the edges of a Sleator–Tarjan Dynamic tree, numbered in the order of installation. This technique was used for quite a prolonged time, until an advanced way of storing edge order, which is shown in the lower right corner of Figure 4, was invented. In the advanced method, the tree edges are connected one after the other by edges of a cyclic graph. For a cyclic graph, it is additionally remembered which edge is the end one. In Figure 4, at the bottom right, the end edge is highlighted in red. The end edge of the cyclic graph points to the first edge of the Sleator–Tarjan Dynamic tree.

In accordance with Figure 4, the cyclic edge interleaving graph and Sleator–Tarjan tree are convenient to be generated using a pair of graphs set on one array. These are an edge interleaving graph and an additional root-node interleaving graph, which, in Figure 4, is a degenerate one and is marked with a gray arrow pointing to itself.

At the beginning of the generation, all nodes are root-nodes. All of them are somehow connected with each other by the graph of the sequence of root-nodes. Then, after merging the first pair of pixels, an edge interleaving graph is created, and the node that has lost its root status is excluded from the root-node interleaving graph, becoming instead the first node of the arc interleaving graph. As the pixel clusters and trees merge, the major root-nodes are connected by edges to the minor nodes. In this case, the major nodes are excluded from the nodes of the root-node interleaving graph and included in the set of nodes of the edge-interleaving graph. Finally, due to the iterative expansion of the trees, N−1 edges appear in the image, and the root-node alternation graph reduces to a graph containing the single node.

It is important that the output edge interleaving graph in a tree is formed in the place of the input image pixels interleaving graph. In order to process any set of pixels as a specific image part by part, for example in terms of Ward’s method, it suffices to use a cyclic graph binding the pixels of this cluster as in Figure 3. Alternatively, a graph of cyclic pointers to the root-nodes of elementary clusters of pixels treated as superpixels is considered as input.

A couple of graphs in Figure 4 provides a fast reproduction of pixel clusters merged in the same order as in the generation stage, but without the accompanying image analysis. For stepwise reproduction of calculations in reverse order, it is enough to invert the edge interleaving graph. Edge-breaking in reverse order in this case defines successive divisions of clusters into two. If online-reverse of calculations is needed, a single-direction edge alternation graph is simply transformed to a *bidirectional* one, containing a pair of graphs of mutually inverse pointers.

**Figure 5:**
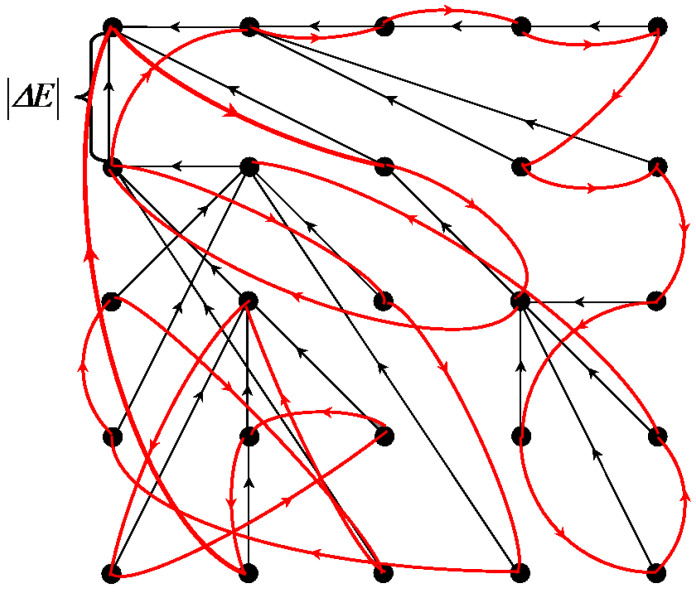
Encoding the hierarchy of pixel clusters with the Sleator–Tarjan Dynamic trees (black lines) and cyclic graphs (red lines) through the example of an image containing 25 pixels.

**Figure 6:**
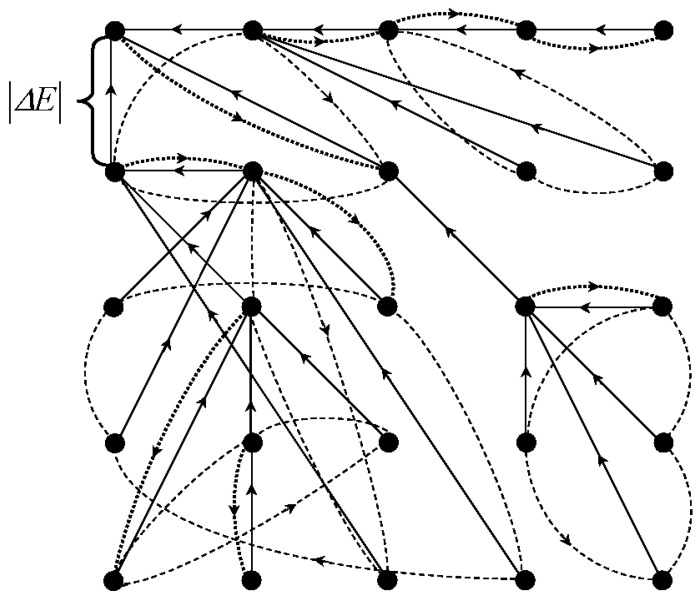
Reversible merge operation through the example of an image containing 25 pixels. The convergent edges of Sleator–Tarjan Dynamic trees (black lines) are interconnected by the edges of the cyclic graphs (dashed lines) in the order in which they were established. The pointers to the edges of the trees, established first, are indicated by dotted lines.

Figure 5 and Figure 6 illustrate the network core of an already generated Algebraic Multi-Layer Network (AMN) data structure using the 25 pixel image as an example.

Figure 5 demonstrates a square image matrix of 25 pixels interconnected by Sleator–Tarjan Dynamic tree edges, which are shown as thin black lines. The tree in Figure 5 has a single root-node, coinciding with the first pixel of the image, which is considered as an identifier for combining all the image pixels into one cluster. When the edge incident to the root-node breaks, the entire tree is divided into two trees, and the set of image pixels is divided into two pixel clusters, which are further considered as sets of pixels of independent images. In addition to the edges of the Sleator–Tarjan Dynamic tree, the image pixels are interconnected by the edges of a cyclic graph, which are shown in Figure 5 with red arched lines and define the order in which the edges of Sleator–Tarjan Dynamic trees are established.

Note that, when applied to cyclic graphs, it is understood that, wherever necessary, they are bidirectional and provide element-wise scanning of pixels in both the forward and reverse order.

Storage of a particular pixel cluster’s characteristics, for example, the heterogeneity value $H\equiv \left|\frac{\partial E}{\partial g}\right|=\left|\Delta E_{split}\right|$, at the address of the edge established at the moment when this cluster was created by merging of two ones is supported by a system of cyclic graphs and pointers, which ensure the ability to locate each next edge at any processing step. The same set of pointers provides reversibility of image pixel clusters merge (Figure 6).

Figure 6 illustrates the matrix of pixels interconnected by edges of a Sleator–Tarjan tree, which are shown as continuous lines. The tree has a single root-node which corresponds to the first pixel and is treated as an identifier for a union of all image pixels merged into a single cluster. At the breaking of the edge incident to the root-node, the tree splits into two ones, and the set of image pixels is divided into two clusters which are further considered as pixels of separate images. For each node in the tree in Figure 6, the incoming edges are combined into cyclic graphs which are shown with dotted lines. Cyclic graphs set an order in which the edges were set up, which is provided with an additional indication for each cycle of either initial or the final node via pointers, shown in bold dotted lines. Cluster merge is specified by setting edges between root-nodes, and a reverse operation of splitting a cluster into two is provided by a break of edges. Under inverting of the pixel cluster merge for a given root-node, a break of edges is performed in reverse order.

It should be noted that, in a reversible merge operation, the rollback modification is supported by the corresponding modification of cycling graphs and Sleator–Tarjan Dynamic trees.

The data structure shown in Figure 6 is defined by three arrays or layers of “neurons”: an array of dynamic trees, an array of cyclic graphs, and an array of pointers to source or destination nodes in cyclic graphs (see also Figure 2).

Sleator–Tarjan Dynamic trees and cyclic graphs (Figure 6) constitute a typical network in which the incoming edges for a particular node are indexed with the values of approximation error increment $H=\left|\Delta E_{split}\right|$, accompanying the bipartition of a pixel cluster defined by this node. The network under consideration is *dynamic*, as it is reconstructed in the process of computation, and is *algebraic*, as it is generated through the merge of trees and cyclic graphs by certain rules. The edge weights $H=\left|\Delta E_{split}\right|$ in Sleator–Tarjan Dynamic trees non-strictly monotonically decrease from the root to the periphery and in the inverted order of establishing the incoming arcs, which expresses the property of the encoded hierarchical approximation sequence to be described by a convex sequence of approximation errors *E*.

Considering modelling of natural neurons, an Algebraic Multi-Layer Network (AMN) includes the following:

- Two or three layers of “neurons” of the network core (Figure 4) containing Sleator–Tarjan trees, and single-directional or bidirectional cyclic graphs of interleaving edges and root-nodes;
- One layer of “neurons” defining pixel alternation in each cluster (Figure 3);
- Four layers of “neurons” containing additive characteristics of clusters in coordinates of root-nodes, including the number of pixels in a cluster and three integral intensities for color components;
- One layer of “neurons” which contain the value $H=\left|\Delta E_{split}\right|$ of heterogeneity for each cluster;
- One layer of “neurons” which define *straightened* trees with pointers to root-nodes;
- Two or three layers of “neurons” providing reversibility of cluster merge (Figure 6), including a layer of pointers to the last edge, and one or two layers of “neurons” with pointers of single-directional or bidirectional cyclic graphs, indicating the order of converging edges.

AMN provides efficient reversible pixel cluster merging with the possibility of modification while reproducing calculations in reverse order. At the low level of calculations, the main programs are a program to merge a pair of pixel clusters and a program to divide a particular pixel cluster in half. The first program accepts a pair of root-nodes at the input which are to be connected to each other to describe cluster merging. For the second program, it suffices to indicate at the input the single root pixel node, matching the cluster which is to be split into two clusters. Depending on the sequence of merge and split instructions for a particular pixel clusters in AMN, a hierarchical image presentation is automatically established and optimized.

Thus, the AMN network is “thrown over” the image pixels, which allows it to develop a specific language to describe sets of pixels in terms of coordinates of pixels themselves. With that, calculations can be arranged without multiple scanning and summation data and also without moving it from place to place. As a result, the software implementation of the discussed methods of *E* minimization with the aid of operations with sets of pixels from any binary cluster’s hierarchy turns out to be comparable in complexity to the programming of single-pixel operations.

## 6. Specifics of Approach to Object Detection

One of the topical problems seems to be a classical problem of optimal data clustering, regarding the confinement of color images to the problem of calculating the series of optimal image approximations within a limited color range, say, from 1 to 100. Optimal (or close to, i.e., *optimal-like*) approximations in 1, 2, …, 100 colors have not been gained yet, even for Lenna or other standard color images. At the same time, the possibilities of classical pixel clustering along with minimization of approximation error *E* have not been developed sufficiently, and the problem is often categorized as almost unresolvable. Insufficient pixel clustering and even less efficient image segmentation at the initial processing stage leads to a more difficult task of selecting objects in an arbitrary image instead of dividing a specific scene image into object images. Where the *selection* of objects is understood as the search for some unique pixel sets without taking into account the pixels that describe the rest of the objects in the same image, but rather taking into account additional a priori data. Thus, the formulation and practical solution of the classical optimization problem of pixel clustering simplifies object disjunction within a limited set of pixel clusters of a given image.

As is known, for grayscale images, the exact solution of the optimization problem is provided by the multi-threshold Otsu method [46], but obtaining optimal image approximations in dozens of colors becomes practically unattainable due to excessive computational complexity.

According to our experience, obtaining practical optimal approximations, at least for grayscale images, turns solvable [20]. For the problem of object detection, the exact solution is helpful but not indispensable. It is much more important that a series of optimal image approximations in different numbers of colors are easier to obtain than any of them separately. This is facilitated by the experimentally established convexity property of the approximation error sequence, which is applicable to any sequence of more than two image approximations.

The paper contains an overview of the hierarchical and optimal approximation model, which has been developed over several decades within the framework of the problem detection of objects in the image. The gradual development of the model has led to the classical clustering algorithms that remove the contrived condition for the mandatory use of a priori data in the stage of initial image processing.

An important feature of the model is the condition of detecting all objects in the scene, and this task is confined to disjunction of the objects without using a priori information, such as learning data.

The model takes into account a dual ambiguity of image, which consists in the fact that, depending on the task being solved, a particular pixel can refer to the following:

- Various objects constituting a hierarchy;
- Various hierarchies of objects.

Therefore, optimal approximations with a different number $g_{0}$ of pixel clusters (colors) originate different hierarchies of objects detected by a computer, where $g_{0}$ is interpreted as the number of *basis* objects to which a computer program is tuned for the best detection of objects-of-interest as basis objects themselves, their unions, or parts of basis objects.

An essential and innovative development in this paper is a plain and accurate definition of superpixels and the hierarchy of superpixels which allows us to move from processing of a traditional pixel matrix to processing of an image consisting of elementary sets of pixels. Defining elementary sets of pixels (superpixels) as intersection of a series of *g* basic optimal image partitions into 1, 2, …, *g* clusters explains the difficulty in detecting objects in agglomerative (bottom up) algorithms of pixel segmentation or pixel clustering. While developing agglomerative algorithms, a programmer has to take into account local, texture characteristics of distribution of close pixels, which are indistinguishable to them, and contend with detection of objects-of-interest, disregarding the characteristics of other objects. In this case, one has to use a priori data on objects-of-interest, and the task confines itself to detecting sets of pixels with pre-set characteristic features. In the discussed model, a less complicated task of disjuncting objects in the image is solved and the above-mentioned problem is overcome with the help of divisive (top-down) algorithms which provide detection of all the objects in the scene but envisage a pre-constructed adaptive hierarchy of image approximations.

As the initial hierarchy of approximations for a color image is constructed by adaptive agglomerative algorithms, a necessity arises in an efficient data structure which supports reversible calculations and optimization of any binary hierarchy of pixel clusters while reproducing calculations in reverse order. Speed operations with millions of approximations and with pixel clusters of a Full HD image in limited RAM are provided by Sleator–Tarjan Dynamic trees and cyclic graphs which, together with other graphs, constitute an Algebraic Multi-Layer Network (AMN, Figure 2) supporting a reversible merge of arbitrary clusters of pixels (Figure 3, Figure 4, Figure 5 and Figure 6). It should be noted that AMN in the model of hierarchical and optimal image approximations does not influence the logic or meaningful interpretation of calculations in any way, but only provides acceleration of calculations and limited usage of operating memory.

The simplest way to implement the model of hierarchical and optimal image approximations without resorting to optimization of calculations is its application to grayscale images. In this case, the calculations are provided by versions of Otsu’s multi-threshold and hierarchical methods [20,46], intended for processing of such images.

In the general case of color images, calculations are provided by three modernized methods of classical cluster analysis: Ward’s method for generation of a hierarchy of approximations, as well as the split/merge CI-method [7] and the K-meanless method (modernized K-means method [7,44]) for the improvement of structured image approximations (Table 1).

In Table 1, the left column describes modernization of standard methods of cluster analysis available in MatLab and other packages. The right column of Table 1 comments on some specifics of software implementation.

When programming image processing according to N. Otsu, modernization involves only the exclusion of histogram normalization, since the latter contradicts the minimization of *E*. In the case of grayscale images, the K-means method can be dispensed with, since it is used to compensate for the inapplicability of Otsu methods in the general case of color images. Modernization of the classical methods of approximation error minimization (Table 1) provides an extension of the capabilities of Ward’s method, and also combined merge/split and K-means methods without worsening the results of their traditional application. Thus, it makes sense to modernize each of the methods separately. At the same time, for the most efficient *E* minimization, it is important to use combinations of modernized methods.

At present, methods of K-means type seem to be the most popular and are actively studied as independent methods for minimizing *E* [40,41,42]. In our opinion, in this case, the experience of inefficient pixel clustering piles up. For hierarchical clustering, dendrograms are usually used, which contributes to an insufficiently adequate description of the hierarchies of pixel sets. Modernization of the classical pixel clustering methods for minimizing the approximation error *E* corrects these shortcomings.

Speed and memory optimizations are achieved by the Algebraic Multi-Layer Network (AMN). In our experience, programming operations with sets of pixels in a specific graph language is beyond the power of an ordinary programmer. Therefore, for AMN implementation, it is necessary not only introduce the modernized methods into software, but also to place them in public application packages such as MatLab.

## 7. Experimental Results

**Figure 7:**
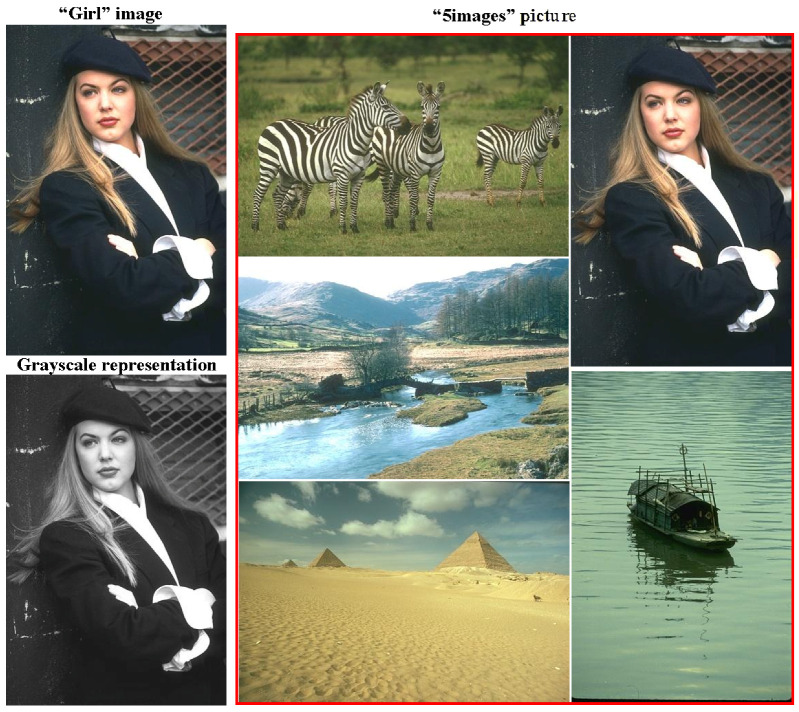
Three sample images for experiments. (**Left**): “Girl” image (**top**) of 321×481 pixels and the corresponding grayscale representation of this image (**bottom**). (**Right**): composite “5images” picture, consisting of five image components, merged into a single entity of 814×978 pixels.

We illustrate some specific aspects of our approach, considering, as examples, the results of image processing. We first examine the images given in Figure 7. These are the images treated in [47].

The paper [47] is conceptually similar to [14], aiming to obtain an adequate image segmentation. The algorithm is designed with regard to the relationships among the adjacent pixels. In contrast to Koepfler’s approach [8], not one single numerical criterion, but rather several are analyzed here to evaluate the quality of a segmented image. There is no requirement for these criteria to reach extreme values simultaneously. It is assumed that, depending on the relevant objects, preference is given to a certain criterion. It is shown that the developed segmentation method outperforms the similar methods in several criteria. This can be attributed to the failure to achieve real optimal values, though, in intermediate calculation steps, Otsu’s multi-threshold method is applied, which ensures an accurate minimization of the approximation error *E* for grayscale images. Contrary to the elementary cluster analysis [15,16], in [47] we obtained as output such versions of segmented images, which did not belong to piecewise constant image approximations with g=1,2,… of colors in the image. Invariance conditions were not regarded here.

Images merged into a single composite in Figure 7 and the Figures below are outlined in red.

Compared to [47], we set up and solved a more generalized problem of invariant image segmentation through pixel clustering here. This setting depends only on the relevant image, i.e., does not change in transformations such as image scaling and converting the image from positive to negative. This setting also remains robust with regard to changes in the image context and in the instance of image conversion to its grayscale representation. To solve the problem at hand, we estimated the optimal image approximations in g=1,2,… colors. Realistic estimation of the optimal image approximations principally do not depend on the actual method the approximations were obtained with, since the definition of the optimal approximation does not limit the methods, available for its generation. However, it is the most straightforward approach to calculate optimal image approximations using classical cluster analysis methods and, primarily, Ward’s pixel clustering method.

Let us estimate the optimal approximations for the color 321×481-pixel “Girl” picture to compare them with the optimal approximations for grayscale “Girl” representation. The condition for adequate object detection should be expressed in the comparability of pixel clustering in color and grayscale representations, since they are perceived as images of the same objects (which they are).

**Figure 8:**
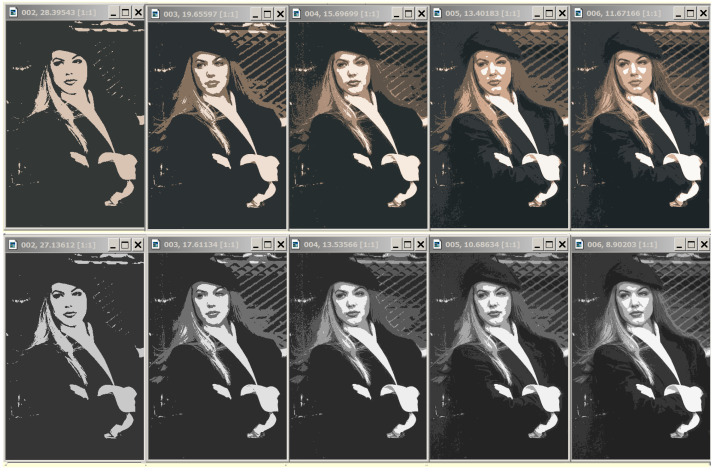
Comparison of optimal approximations for color and grayscale image representations. In the upper row, the optimal color image approximations in g=2,3,4,5, and 6 colors are presented from left to right, and, below them, similar approximations of the grayscale image are shown. Image approximations at the top are labeled with *g* cluster numbers and standard deviation values σ.

In Figure 8, the grayscale image representations in 2, 3, and 4 intensity levels from the bottom row look like they were obtained by transforming the above color image representations in 2, 3, and 4 colors. In optimal approximations in 5 and 6 gradations, several light flecks appear differently in color and grayscale representations.

**Figure 9:**
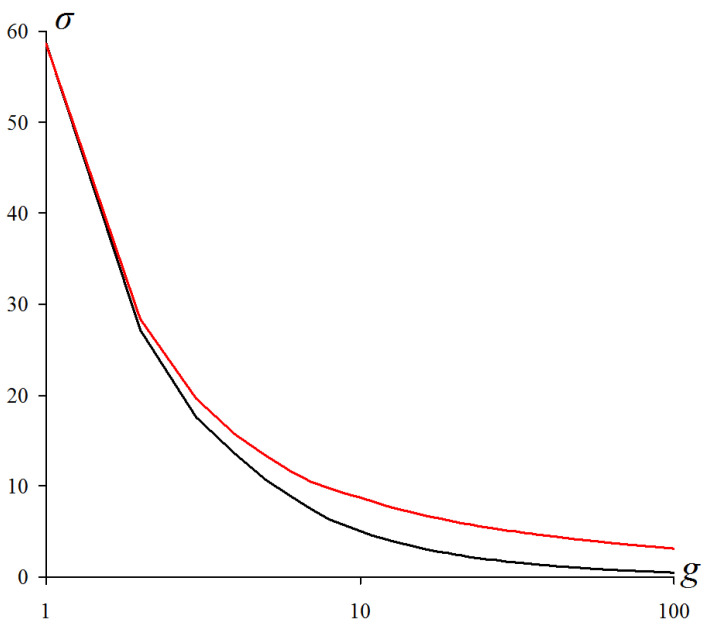
Comparison of optimal approximations for color and grayscale image representations. Minimal standard deviations for the number of cluster numbers ranging from 1 to 100. The top red graph describes a color image and the bottom black graph describes a grayscale representation.

Figure 9 shows the dependences of the standard deviation σ on the number *g* of cluster numbers in the range from 1 to 100 for optimal approximations of the color and grayscale image representations.

Characteristically, both curves in Figure 9 are strictly convex. The difference between the curves seemed to be related to the change in the operating intensity range.

**Figure 10:**
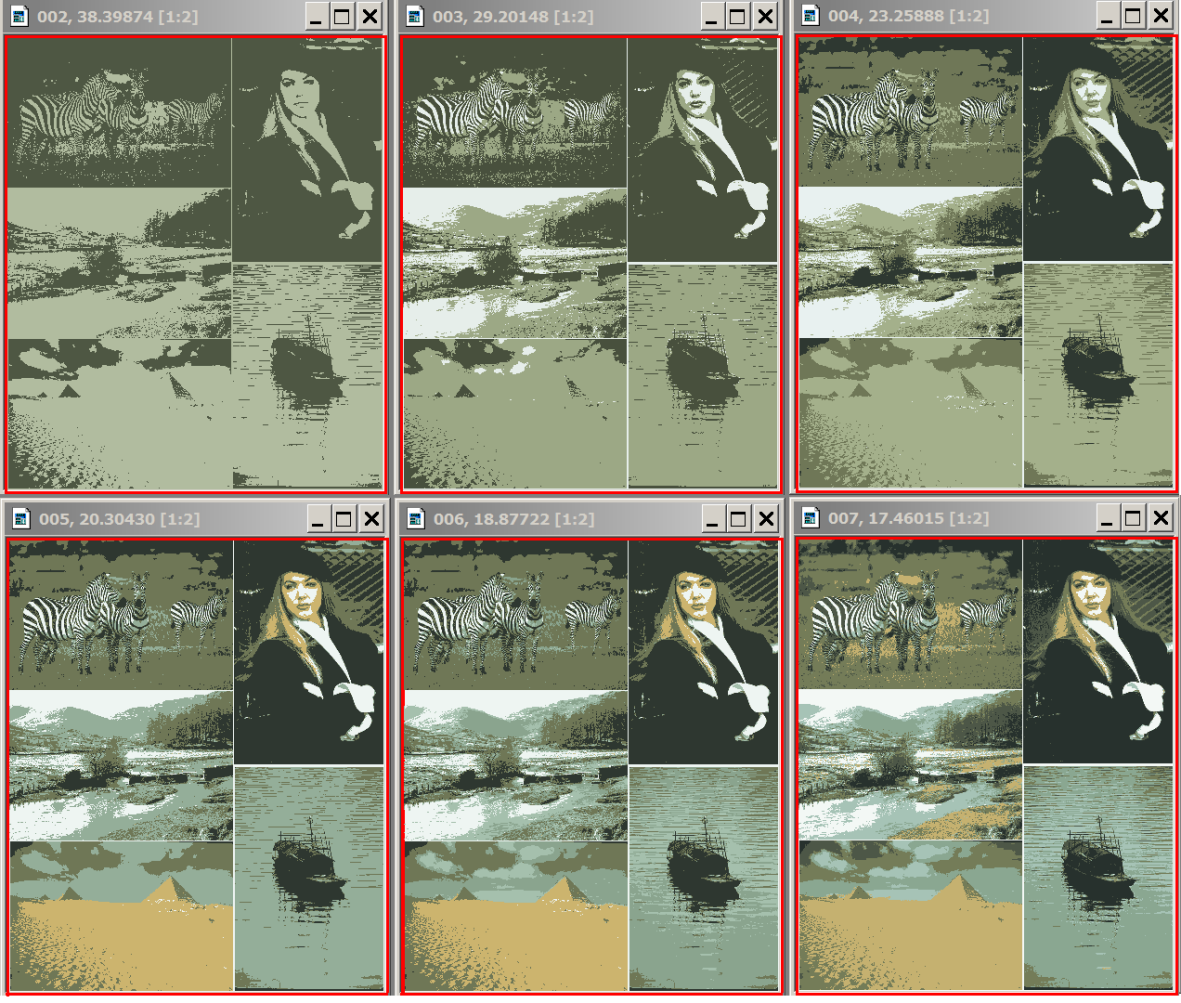
Optimal approximations of “5images” picture in two to seven colors arranged in two rows in lexicographic order. Image approximations at the top are labeled with *g* color numbers and standard deviation values σ.

As it turned out, there was a similar effect of stable segmentation of the object, regardless of the image content. This effect is illustrated in (Figure 10).

Figure 10 shows the optimal approximations in two to seven colors for the 814×978-pixel “5images” picture (compare the optimal approximations for the 321×481-pixel “Girl” image, given in Figure 8 with the optimal approximations of the same image embedded into the “5images” picture in Figure 10). The observed stability effect can be, to some extent, explained as follows: optimal pixel clustering is achieved via image merging, both in the case of merging identical images and when merging such images, where the average intensities turn out to be quite disparate, although the intensity ranges may overlap.

In general, objects in a given image are divided into stably and unstably segmented ones. This seems promising for object classification and can provide a separation of foreground and background objects, automation of artistic photography, and simpler handling of other applied problems.

The similarity of optimal approximations of an image in color and grayscale representations can be utilized in different ways. Since, compared to color images, optimal partitions are much easier to calculate for grayscale representations, they can be taken as initial ones when calculating optimal approximations for color images.

As far as purely engineering applications are concerned, the study of the relationship between optimal approximations of a color image and grayscale representation is obviously useful for solving colorization problems [48], avoiding heuristic learning.

It should be noted that the learning process involves human participation and does not seem necessary when modeling the vision of simpler systems, say, insects. The primary ordering of the input data by the number of colors *g*, the approximation error *E*, and the heterogeneity parameter $H=\left|\Delta E_{split}\right|$ is treated here as a common stage of perception for different natural visual systems. Data ordering can be represented as a program that automatically generates the so-called Dynamic Table of image approximations introduced in [7].

**Figure 11:**
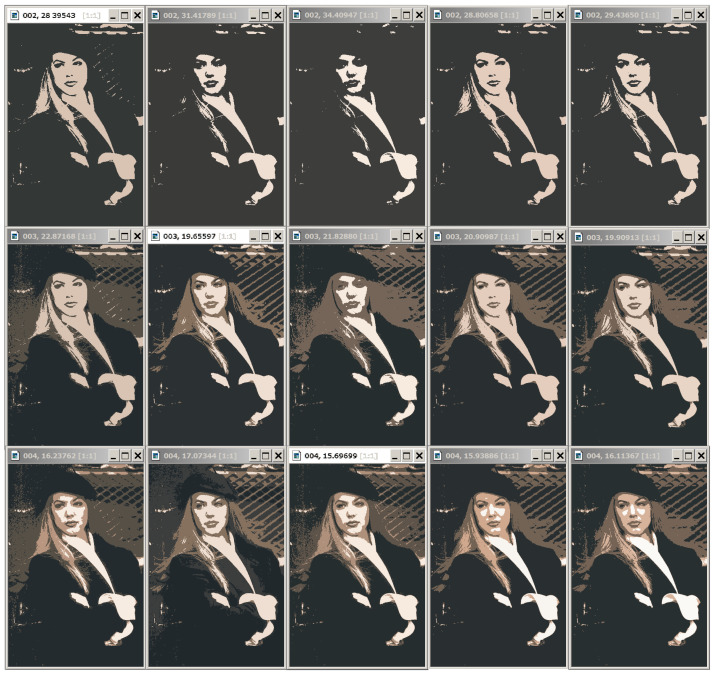
Dynamic Table of approximations for “Girl” color image (dimensions 321×481 pixels). The first row and first column of the table were cropped. The columns of Dynamic Table, containing the optimal image approximations in 2–6 colors, are shown. Each column contains a binary hierarchical sequence of image approximations with incrementally added colors: g=2,3, and 4. On the main diagonal of the Dynamic Table are the optimal image approximations in $g_{0}=2,3$, and 4 colors. Image approximations at the top are labeled with *g* color numbers and corresponding standard deviation values σ.

The Dynamic Table for “Girl” image is shown in Figure 11.

Figure 11 shows the fragment of the Dynamic Table that actually illustrates Figure 1. Hierarchies of image approximations are located in columns of the Dynamic Table. When the row number is increased by 1, one of the colors in the current image approximation is split up into two ones. The diagonal approximations are just that in the upper row of Figure 8, which are improved in the error *E* when applying various generation algorithms. Parameter $g_{0}$ is equal to the number of colors in the optimal approximation of the image and is counted along the diagonal.

In the user’s view, the entire Dynamic Table of N×N image approximations is allocated in RAM. In fact, it is encoded in RAM by AMN core (Figure 2 and Figure 5) and the viewed approximations are generated on-line as needed. That is why the discussed table is called a *Dynamic Table*.

The user’s task is to choose a column of approximations in which the structured objects-of-interest are best displayed. According to the user’s choice, the tuning parameter $g_{0}$ is set up, and the objects are approximated either by unions or by parts of pixel clusters of the optimal image approximation in $g_{0}$ colors. For example, in the problem of identifying a person by the relative position of the eyes, corners of the lips, and the tip of the nose, the penultimate hierarchy of image approximations and the corresponding parameters $g_{0}=2$ and 3 are preferable.

A Dynamic Table contains a sequence of hierarchies of image approximations. Given optimal image approximations, it can be easily generated using Ward’s pixel clustering method, as described in Section 2. However, optimal approximations as such may be poorly suited for detecting some objects, since certain sharp boundaries separating pixel clusters are erased in the non-hierarchical image structure, but they can remain in optimal approximations, where the color range is much narrower.

Such boundaries are relevant in superpixel image approximations in 1,2,…,k(i),…,N colors, where i=1,2,3,…,N, i≤k≤min(i!,N), and exclamation mark denotes factorial. To take into account every one of the sharp boundaries, it is sufficient to build hierarchies of image approximations using the original Ward’s method, starting from superpixel approximations. In this case, the superpixels are considered as unit objects, and the rest objects are obtained by iterative merging of the superpixels.

**Figure 12:**
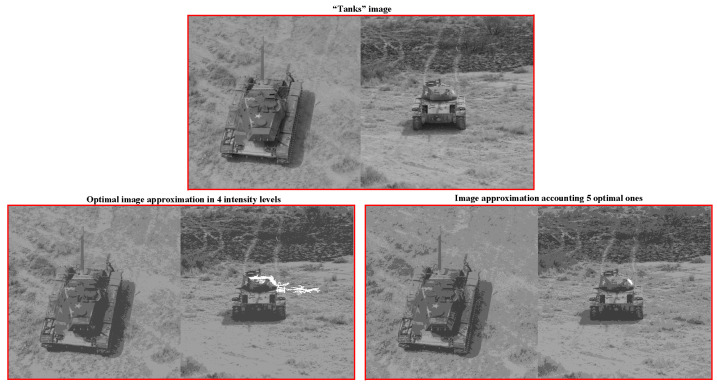
Improving object detection using superpixel approximations. (**Top**): Sample images from the SIPI Image Database combined into a single 1024 × 512-pixel “Tanks” image. (**Bottom**): Optimal image approximation in four intensity levels (**left**) and improved image approximation in four intensity levels (**right**). The segments containing the object-of-interest are filled with white.

Figure 12 illustrates how the optimal image approximations can be improved.

Figure 12 shows two images of tanks, combined into a single entity in order to perform object detection without performing any feature analysis or identification. Let us consider as relevant objects the three five-pointed stars on the tank armor. These five-pointed stars have to be highlighted in the same intensities. For the rightmost five-pointed star, this is more difficult than for the rest ones because it melts into the background, as shown in the bottom left image approximation. The relevant five-pointed star considered here has been successfully detected in the bottom right image approximation.

The bottom right image approximation in four intensity levels is obtained by intersecting the first five optimal image approximations, followed by iterative merging of the resultant eleven pixel clusters down to four. At the same time, generating the optimal image approximation in four intensity levels results in the destruction of the problematic object, as shown at the bottom left of Figure 12. Hence, it is interesting to provide this at the output of image structuring, involving the generation of a so-called Dynamic Superpixel Table, which, in practical terms, is used in the same way as the Dynamic Table demonstrated above.

The *i*-th column of Dynamic Superpixel Table is generated by Ward’s clustering of superpixels of the i-th superpixel approximation, which is obtained by intersecting the optimal image approximations in 1,2,…,i intensity levels. If the image turns out to be hierarchically structured, then the superpixel approximations coincide with the optimal ones, and the Dynamic Superpixel Table coincides with the upper triangular approximation table, cut off by the main diagonal of Dynamic Table. Otherwise, the number k(i) of approximations in the *i*-th column of the Dynamic Superpixel Table exceeds *i*.

**Figure 13:**
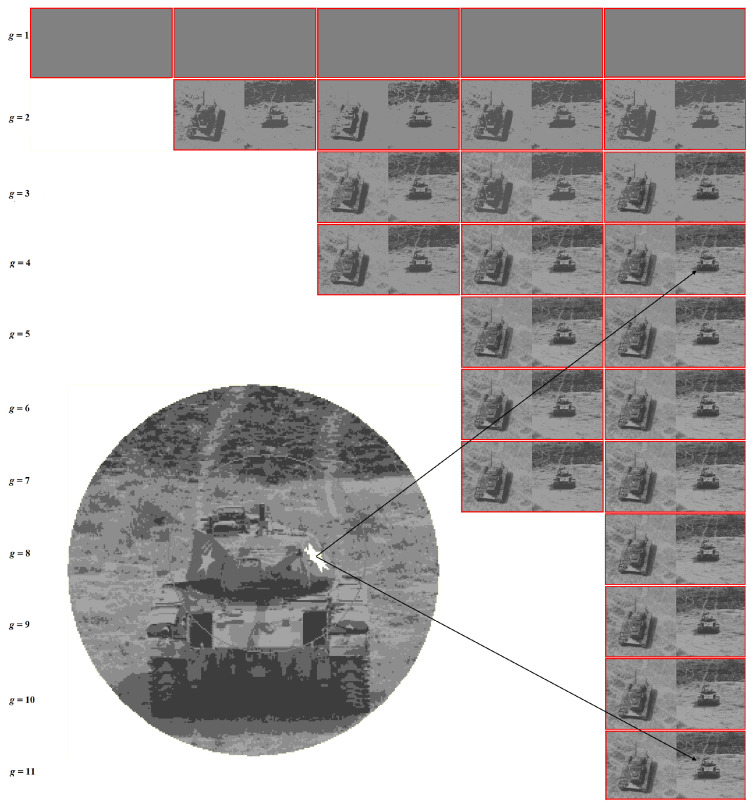
Dynamic Superpixel Table for the grayscale “Tanks” image (dimensions 1024×512 pixels). The columns contain binary hierarchical sequences of approximations in the incremental number of intensity levels from g=1 to g=11, indicated on the left. The lowest approximations in each column are the superpixel approximations obtained as intersections of the 1,2,… optimal image approximations. The other approximations are generated from the latter using Ward’s pixel clustering. A problematic five-pointed star (filled with white) appears at approximations in intensity levels 4–11.

Dynamic Superpixel Table for the grayscale “Tanks” image is shown in Figure 13.

In practical terms, it may be useful to a priori define objects-of-interest as superpixels that persist in a succession of image approximations in an incrementally increasing number of colors or grayscale intensity gradations.

Regarding the Dynamic Table and the Dynamic Superpixel Table at the output of the image structuring, it should be noted that they are generated completely automatically, which is extremely important. Less important is the generation rate. Of the many ways to boost calculations, it is necessary to choose those that do not introduce coupling of calculations to images of specific content. For this, it is necessary to make sure, in advance, that the calculation routine is adequate for the problem at hand to obtain reference values for control purposes.

The “Tanks” image optimal approximations of 1024×512 pixels and “Girl” grayscale optimal approximations of 321×481 pixels were obtained as in [20]. The optimal approximations of the “Girl” color image and “5images” color picture of 814×978 pixels were obtained by many iterations of Ward’s method, using different initial values of enlarged pixel count, with further selection of approximations having the lowest values of error *E* for each color number, ranging from 1 to 100. For this purpose, Ward’s method was run by two versions of the software implementation, with the number $\tilde{N}$ of enlarged pixels in the range from 100,000 to 154,401 for the “Girl” color image and with $\tilde{N}$ in the range from 560,000 to 796,092 for the “5images” color picture.

## 8. Conclusions

As is known, the forced use of learning or a priori known information about objects in the image complicates the problem [49]. To overcome these obstacle, we relied on classical cluster analysis methods, modernized them for modern applications, and formalized the concepts of an image, superpixels, and object hierarchy through optimal image approximations. The only difficulty came down to the organization of high-speed calculations.

There are many ways to speed up calculations.

For example, acceleration is achieved by any of the following:

- Apply Ward’s method within the image parts, followed by the CI method of processing;
- Enlarge the original pixels, replacing them with enlarged image segments or superpixels;
- Reduce the number of colors in the image within the limits of visual invisibility.

The first method is preferable, since it does not use modifications to the original data. This method allows for various implementations that need to be explored. The second method works in current software implementations. The third method should be used with caution, as it suppresses the variability of Ward’s method.

However, it seems obvious thatm for an acceptable speed of processing an image of *N* pixels by Ward’s method, an insurmountable obstacle is the need to scan $N^{2}$ pairs of pixels (and the more interesting it is to deal with this problem). The idea is to represent the image as multispectral by using pixel decimation.

Pixel decimation is an operation that models the inverse operation of pixel duplication commuting with pixel clustering by the methods in question. However, pixel decimation results in several reduced-sized images. In order not to lose data, the idea arises of combine these several images with several of increased pixel dimensions. Programming this will not be difficult at all. We hope to present the results in our next paper.

## 9. Patents

There is a patent resulting from the work described in this manuscript: Nenashev, V.A.; Khanykov, I.G., and Shepeta, A.P. Device for multiple-angle synthesis of complex image of the Earth’s surface, Patent for invention RU 2756904 C1, 10/06/2021. Application No. 2021107671 dated 08/24/2020, 15p, 2021.

## Figures and Tables

**Table 1:** Modernization of classical methods of cluster analysis.

Computing Technique	Specifics
Otsu’s multi-threshold and hierarchical methods	Only for grayscale images, without normalizing of histogram
Ward’s method → recursive Ward’s method	Speed-up image processing by parts
Split/merge methods → CI-method	Reversible merging pixel clusters
K-means method → K–meanless method	Exact criterion for reclassification of cluster parts
Dendrograms → AMN	Working with pixel clusters via pixel coordinates

## Data Availability

The data set used for experimentation in this study is taken from BSDS500 and USC-SIPI, and these data are available in the public domain. Those used in the paper and other useful program codes are available at: https://disk.yandex.ru/client/disk/ExperimentalFindingsKhar (accessed on 2 April 2023). Requests for advice on the operation of programs are welcome.

## References

[1] Nawaz M., Yan H. (2020). Saliency Detection using Deep Features and Affinity-based Robust Background Subtraction. IEEE Trans. Multimed..

[2] Fareed M.M.S., Ahmed G., Chun Q. (2015). Salient region detection through sparse reconstruction and graph-based ranking. J. Vis. Commun. Image Represent..

[3] Mishro P.K., Agrawal S., Panda R., Abraham A. (2021). A Novel Type-2 Fuzzy C-Means Clustering for Brain MR Image Segmentation. IEEE Trans. Cybern..

[4] Bora D.J., Gupta A.K. (2014). Clustering approach towards image segmentation: An analytical study. arXiv.

[5] Chuang K.S., Tzeng H.L., Chen S., Wu J., Chen T.J. (2006). Fuzzy c-means clustering with spatial information for image segmentation. Comput. Med. Imaging Graph..

[6] Pappas T.N., Jayant N.S. (1989). An adaptive clustering algorithm for image segmentation. Int. Conf. Acoust. Speech Signal Process..

[7] Nenashev V.A., Khanykov I.G., Kharinov M.V. (2022). A Model of Pixel and Superpixel Clustering for Object Detection. J. Imaging.

[8] Koepfler G., Lakshmikantham V. (1996). Segmentation by minimizing functionals and the merging methods. World Congress of Nonlinear Analysts’92, Proceedings of the First World Congress of Nonlinear Analysts, Tampa, FL, USA, 19–26 August 1992.

[9] Mumford D., Shah J. Boundary detection by minimizing functionals. Proceedings of the IEEE Conference on Computer Vision and Pattern Recognition.

[10] Mumford D.B., Shah J. (1989). Optimal approximations by piecewise smooth functions and associated variational problems. Commun. Pure Appl. Math..

[11] Julesz B. (1986). Texton gradients: The texton theory revisited. Biol. Cybern..

[12] Malik J., Perona P. A computational model of texture segmentation. Proceedings of the Twenty-Second Asilomar Conference on Signals, Systems and Computers.

[13] Bugaev A.S., Khelvas A.V. (2001). Exploratory Research and Development of Methods and Tools for Analysis and Automatic Recognition of Streaming Information in Global Information Systems. Cipher “Latskan”.

[14] Wang Z., Bovik A.C., Sheikh H.R., Simoncelli E.P. (2004). Image quality assessment: From error visibility to structural similarity. IEEE Trans. Image Process..

[15] Aivazian S.A., Bukhshtaber V.M., Eniukov I.S., Meshalkin L.D. (1989). Prikladnaia Statistika: Klassifikatsiia i Snizhenie Razmernosti [Applied Statistics: Classification and Dimension Reduction].

[16] Mandel I.D. (1988). Klasternyi Analiz [Cluster Analysis].

[17] Murtagh F., Pierre L. (2014). Ward’s hierarchical agglomerative clustering method: Which algorithms implement Ward’s criterion?. J. Classif..

[18] Torrente A., Romo J. (2021). Initializing k-means Clustering by Bootstrap and Data Depth. J. Classif..

[19] Williamson D.P., Shmoys D.B. (2011). The Design of Approximation Algorithms.

[20] Kharinov M.V. (2013). Image Segmentation Method by Merging and Correction of Sets of Pixels. Pat. Recog. Image Anal. Adv. Math. Theory Appl..

[21] Murtagh F., Legendre P. (2011). Ward’s hierarchical clustering method: Clustering criterion and agglomerative algorithm. arXiv.

[22] Fernández A., Gómez S. (2019). Versatile Linkage: A Family of Space-Conserving Strategies for Agglomerative Hierarchical Clustering. J. Classif..

[23] Tokuda E.K., Comin C.H., Costa L.D.F. (2022). Revisiting agglomerative clustering. Phys. A Stat. Mech. Its Appl..

[24] Ward J.H. (1963). Hierarchical grouping to optimize an objective function. J. Am. Stat. Assoc..

[25] Toffoli T. (1980). Reversible computing. International Colloquium on Automata, Languages, and Programming.

[26] Zongxiang Y. (2011). Reversible Three-Dimensional Image Segmentation. U.S. Patent.

[27] Sleator D.D., Tarjan R.E. (1985). Self–Adjusting Binary Search Trees. J. ACM.

[28] Nock R., Nielsen F. (2004). Statistical Region Merging. IEEE Trans. Pattern Anal. Mach. Intell..

[29] Gurevich I., Yashina V. (2021). Basic models of descriptive image analysis in Pattern Recognition. Proceedings of the ICPR International Workshops and Challenges.

[30] Kharinov M. Example-Based Object Detection in the Attached Image. Proceedings of the Graphicon-Conference on Computer Graphics and Vision.

[31] Tran T.N., Wehrens R., Buydens L.M.C. (2003). SpaRef: A clustering algorithm for multispectral images. Anal. Chim. Acta..

[32] Owen S.M., MacKenzie A.R., Bunce R.G.H., Stewart H.E., Donovan R.G., Stark G., Hewitt C.N. (2006). Urban land classification and its uncertainties using principal component and cluster analyses: A case study for the UK West Midlands. Landsc. Urban Plan..

[33] Lasch P., Haensch W., Naumann D., Diem M. (2004). Imaging of colorectal adenocarcinoma using FT-IR microspectroscopy and cluster analysis. Biochim. Biophys. Acta (BBA)-Mol. Basis Dis..

[34] Thirion B., Varoquaux G., Dohmatob E., Poline J.B. (2014). Which fMRI clustering gives good brain parcellations?. Front. Neurosci..

[35] Bali A., Singh S.N. A Review on the Strategies and Techniques of Image Segmentation. Proceedings of the 2015 Fifth International Conference on Advanced Computing &Communication Technologies.

[36] Ju R.Y., Lin T.Y., Chiang J.S., Jian J.H., Lin Y.S. Aggregated Pyramid Vision Transformer: Split-transform-merge Strategy for Image Recognition without Convolutions. Proceedings of the 2022 IEEE International Conference on Consumer Electronics-Taiwan.

[37] Kharinov M.V., Khanykov I.G. (2015). Optimization of piecewise constant approximation for segmented image. SPIIRAS Proc..

[38] Jain A.K. (2010). Data clustering: 50 years beyond K-means. Pattern Recognit. Lett..

[39] Blömer J., Lammersen C., Schmidt M., Sohler C. (2016). Theoretical analysis of the k-means algorithm—A survey. Algorithm Engineering: Selected Results and Surveys.

[40] Fränti P., Sieranoja S. (2019). How much can k-means be improved by using better initialization and repeats?. Pattern Recognit..

[41] Yang M., Sinaga K.P. (2019). A Feature-Reduction Multi-View k-Means Clustering Algorithm. IEEE Access.

[42] Zhang L., Qu J., Gao M., Zhao M. Improvement of K-means algorithm based on density. Proceedings of the 2019 IEEE 8th Joint International Information Technology and Artificial Intelligence Conference (ITAIC).

[43] Aloise D., Damasceno N.C., Mladenović N., Pinheiro D.N. (2017). On strategies to fix degenerate k-means solutions. J. Classif..

[44] Dvoenko S.D. Meanless k-means as k-meanless clustering with the bi-partial approach. Proceedings of the 12th International Conference on Pattern Recognition and Information Processing (PRIP’2014).

[45] Liu T., Seyedhosseini M., Tasdizen T. (2016). Image segmentation using hierarchical merge tree. IEEE Trans. Image Process..

[46] Ping-Sung L., Tse-Sheng C., Pau-Choo C. (2001). Algorithm for Multilevel Thresholding. J. Inf. Sci. Eng..

[47] Rangu S., Veramalla R., Salkuti S.R., Kalagadda B. (2023). Efficient Approach to Color Image Segmentation Based on Multilevel Thresholding Using EMO Algorithm by Considering Spatial Contextual Information. J. Imaging.

[48] Cheng Z., Yang Q., Sheng B. Deep Colorization. Proceedings of the IEEE International Conference on Computer Vision (ICCV).

[49] Chochia P.A. (2016). Theory and Methods of Video Information Processing Based on a Two-Scale Image Model. Post Ph.D. Thesis.

